# Tumor Suppressor Protein p53 Recruits Human Sin3B/HDAC1 Complex for Down-Regulation of Its Target Promoters in Response to Genotoxic Stress

**DOI:** 10.1371/journal.pone.0026156

**Published:** 2011-10-20

**Authors:** Nidhi Bansal, Rama Kadamb, Shilpi Mittal, Leena Vig, Raisha Sharma, Bilikere S. Dwarakanath, Daman Saluja

**Affiliations:** 1 Dr. B. R. Ambedkar Center for Biomedical Research, University of Delhi, Delhi, India; 2 Institute of Nuclear Medicine and Allied Sciences, Delhi, India; University of Illinois at Chicago, United States of America

## Abstract

Master regulator protein p53, popularly known as the “guardian of genome” is the hub for regulation of diverse cellular pathways. Depending on the cell type and severity of DNA damage, p53 protein mediates cell cycle arrest or apoptosis, besides activating DNA repair, which is apparently achieved by regulation of its target genes, as well as direct interaction with other proteins. p53 is known to repress target genes via multiple mechanisms one of which is via recruitment of chromatin remodelling Sin3/HDAC1/2 complex. Sin3 proteins (Sin3A and Sin3B) regulate gene expression at the chromatin-level by serving as an anchor onto which the core Sin3/HDAC complex is assembled. The Sin3/HDAC co-repressor complex can be recruited by a large number of DNA-binding transcription factors. Sin3A has been closely linked to p53 while Sin3B is considered to be a close associate of E2Fs. The theme of this study was to establish the role of Sin3B in p53-mediated gene repression. We demonstrate a direct protein-protein interaction between human p53 and Sin3B (hSin3B). Amino acids 1–399 of hSin3B protein are involved in its interaction with N-terminal region (amino acids 1–108) of p53. Genotoxic stress induced by Adriamycin treatment increases the levels of hSin3B that is recruited to the promoters of p53-target genes (*HSPA8*, *MAD1* and *CRYZ*). More importantly recruitment of hSin3B and repression of the three p53-target promoters upon Adriamycin treatment were observed only in p53^+/+^ cell lines. Additionally an increased tri-methylation of the H3K9 residue at the promoters of *HSPA8* and *CRYZ* was also observed following Adriamycin treatment. The present study highlights for the first time the essential role of Sin3B as an important associate of p53 in mediating the cellular responses to stress and in the transcriptional repression of genes encoding for heat shock proteins or proteins involved in regulation of cell cycle and apoptosis.

## Introduction

The *p53* gene is widely recognized as the master regulator of diverse cellular networks. p53 is a sequence specific transcription factor capable of transactivation and transrepression [1]–[3]. Although the mechanisms of p53 mediated gene activation are extensively analyzed (reviewed in ref. [4]), bonafide transcription repression by p53 had initially received less attention. The last decade, however, has witnessed identification of principally three mechanisms for repression of a repertoire of p53 target genes: competition with transcription activator for DNA binding, sequestration of transcription activators or recruitment of co-repressor/chromatin-modifying factors (reviewed in ref. [5]). Recruitment of co-repressor complex like Sin3/HDAC complex by DNA binding transcription factor is an evolutionary conserved mechanism of transrepression. Sin3 has been established as a master transcriptional scaffold and co-repressor capable of transcriptional silencing via associated HDACs. In 1999, Murphy and co-workers reported that p53 interacts with mSin3A and negatively regulates two cytoskeletal genes: *Map4* and *Stathmin* [6]. Subsequently various p53 responsive genes like *Mad1*, *HSP90β and Nanog* have been reported to be repressed by p53 via recruitment of Sin3A/HDAC complex to the p53 response element [7]–[9]. In mammals two highly homologous isoforms, Sin3A and Sin3B have been reported [10]. However the functional redundancy and/or specificity of Sin3A and Sin3B are poorly understood (reviewed in ref. [11]). Several studies implicate that these two proteins target similar subset of genes while other reports highlight a clear functional demarcation between the two proteins. At the level of protein-protein interaction MAD1, KLF, REST, ESET interact with both the isoforms while proteins like SMRT and MeCP2 appear to bind specifically to Sin3A [12]–[14]. On the other hand, CIITA mediates its transrepression functions via exclusively recruiting the Sin3B/HDAC2 complex [15]. Sin3A is involved in mediating p53 dependent gene repression [6], [8], while Sin3B/HDAC co-repressor complex is recognized to be an essential regulator of chromatin modification at the E2F-target promoters ([16], reviewed in ref. [17]). The existence of such functional differences/similarities between Sin3A and Sin3B prompted us to investigate whether p53 utilizes Sin3B/HDAC co-repressor for mediating its transrepression function at subset of its target promoters. We demonstrate recruitment of the human Sin3B/HDAC1 complex at three of the p53-repressed target promoters accompanied by altered histone methylation and a concomitant repression of these genes under conditions of genotoxic insult, thereby highlighting for the first time, Sin3B as an important player in p53-mediated gene repression.

## Results

### Human p53 co-immunoprecipitates phosphorylated human Sin3B

**Figure 1:**
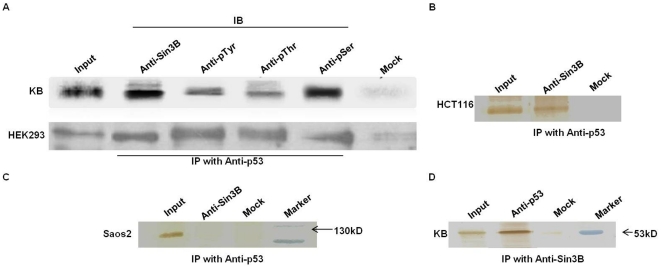
Phosphorylated hSin3B associates with hp53 *in vivo*. (A & B) Cell lysates from KB, HEK293 (A) and HCT116 cell lines (B) were immunoprecipitated (IP) with antibody specific for p53 followed by immunoblot analysis (IB) with antibodies specific for hSin3B (sc-13145 for KB and HCT116 cell lines; sc-55516 for HEK293 cells), phosphorylated serine (anti-pSer), phosphorylated tyrosine (anti-pTyr), phosphorylated threonine (anti-pThr) as indicated above each lane. Western analysis indicates the co-immunoprecipitation of phosphorylated hSin3B with p53 in KB, HEK293 and HCT116 cell extracts. (C) IP-Western analysis in p53-null cell line (Saos2) shows that hSin3B was detectable only in the input lane but not in the immune complex obtained from antibody against p53 or in the mock immunoprecipitates. (D) Reciprocal IP-Western analysis in KB cell extract using the ImmunoCruz™ IP/WB Optima E System (Santa Cruz) as described in the methods section reveals the presence of p53 in a complex with hSin3B. In all the experiments input corresponds to 10% of the total cell lysate used for each immunoprecipitation.

To investigate the role of human Sin3B (hSin3B) in p53 trans-repression functions, we initially performed co-immunoprecipitation assays to test the association between p53 and hSin3B in three different human cell lines. Total cell lysates from wild-type p53^+/+^ cell lines (KB, HCT116 and HEK293) were immunoprecipitated with anti-p53 antibody and the immunoprecipitates were probed for the presence of hSin3B by immunoblot analysis. As shown in figure 1 hSin3B was recovered in the p53 immunoprecipitates in p53^+/+^ cells but not in either the mock immunoprecipitation or the p53-null cell line, Saos2. Keeping in mind the high degree of homology between Sin3A and Sin3B, the identity of the 130 kD band of hSin3B was further examined by western analysis using antibodies targeted against regions specific to Sin3B (antibodies sc-768; sc-55516; sc-13145; Santa Cruz Biotechnology, USA). Identical and reproducible results were obtained with all the three immunoblot analysis suggesting that the 130 kD protein, co-immunoprecipitated with p53, is indeed hSin3B (Figure 1 and Supplementary Figure S1).

Reciprocal IP-Western experiments in KB cells demonstrated that hSin3B could also co-immunoprecipitate p53 protein (53 kD), reaffirming that hSin3B interacts with p53 *in vivo* (Figure 1D). Sin3B has potential sites for several post translational modifications like myristoylation and phosphorylations (reviewed in ref. [11]). The phosphorylation status of p53-bound Sin3B was analyzed by immunoblotting p53 immune complexes with antibodies targeted against phosphorylated serine, threonine and tyrosine. Appearance of a phosphorylated 130 kD protein band juxtaposed to the hSin3B protein (Figure 1A), observed with all the three antibodies, suggested that hSin3B interacting with p53 is indeed phosphorylated. However, whether phosphorylation is crucial for this interaction and the role of phosphorylation, if any, in mediating p53-Sin3B functions remain to be elucidated.

Our studies show that hSin3B co-immunoprecipitates with HDAC1 in a p53-independent manner (see Supplementary Figure S2). This is consistent with the previous reports showing that Sin3B mediates the trans-repression function of various DNA-binding transcription factors via interaction with HDAC1/2 [16], [18], [19]. Taken together these results suggest that p53 can utilize Sin3B/HDAC1 complex for its transrepression functions.

### Paired Amphipathic Helices (PAH) 1–3 domains of hSin3B are crucial for direct protein-protein interaction with human p53

**Figure 2:**
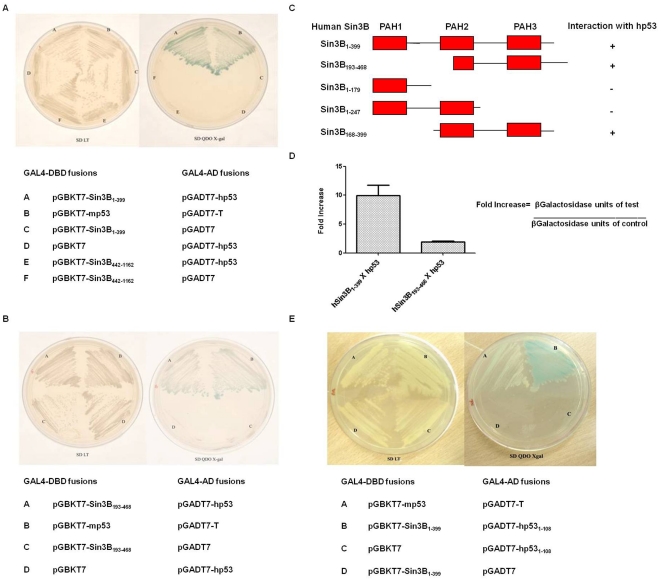
Yeast two Hybrid analysis for the interaction of hSin3B with hp53. (A) & (B) Yeast AH109 cells were co-transformed with plasmids indicated below the plates for each sector. Successful co-transformations were confirmed by growth on SD LT plates (Drop-out medium lacking Leucine and tryptophan). The protein-protein interactions were checked by growing the co-transformants on selective SD QDO-Xgal medium (Quadruple drop-out medium lacking leucine, tryptophan, adenine and histidine and containing X-gal). Positive interaction was observed only between pGBKT7-Sin3B_1–399_ and pGADT7-hp53 (Figure 2A, sector A) as well as pGBKT7-Sin3B_193–468_ and pGADT7-hp53 (Figure 2B, Sector A). (C) Schematic representation of the various truncated forms of hSin3B used in the yeast two hybrid assays. Each truncated Sin3B construct was co-tranformed with hp53 in AH109 cells and interaction was checked by observing growth on selective medium (SD QDO-Xgal). A plus sign (+) indicates positive interaction and negative sign (−) indicates no interaction. (D) β-galactosidase assays were performed to quantify two-hybrid interactions. A 9.9±1.813 fold increase in the relative β-galactosidase units was observed for hp53/Sin3B_1–399_ interactions while a 1.9±0.107 fold increase was observed for hp53/Sin3B_193–468_ interaction. All values are plotted with ±SEM calculated for three independent experiments. (E) Yeast AH109 cells were co-transformed with plasmids indicated below the plates for each sector. Positive interaction was observed between pGBKT7-Sin3B_1–399_ and pGADT7-hp53_1–108_ (sector B) as indicated by growth on selective medium (SD QDO-Xgal).

To confirm direct protein-protein interaction between hSin3B and human p53 (hp53), yeast two hybrid assays were performed. Each of the three overlapping fragments spanning full length coding sequence of hSin3B were fused to the GAL4 DNA binding domain and were used as bait (Supplementary Figure S3). Human p53 was fused with GAL4 activation domain and was used as prey. All the yeast two hybrid specificity controls did not autonomously activate the reporter genes. AH109 cells of *Saccharomyces cerevisiae* co-transformed with pGBKT7-p53 (murine p53) and pGADT7-T (Large T antigen) were used as positive control. AH109 cells, co-transformed with hp53 and hSin3B _1–399_ (spanning PAH 1–3 domains of hSin3B) or hSin3B _193–468_ (spanning PAH 2–3 domains of hSin3B) could grow and produce blue colonies on quadruple drop-out medium with X-gal (QDO-Xgal; Figure 2A and B), whereas cells co-transformed with hp53 and pGBKT7-Sin3B_442–1162_ or vector alone did not grow on QDO-Xgal plates suggesting that Sin3B _442–1162_ (spanning HID, PAH4 and HCR domain of hSin3B; Supplementary Figure S3) was not capable of interaction with hp53. The lack of growth on selection medium was not due to lack of expression of hSin3B_442–1162_ as all the clones of hSin3B expressed at detectable levels (Supplementary Figure S4). Three additional truncated hSin3B constructs (Sin3B_1–247_, Sin3B_1–179_, Sin3B_168–399;_ Figure 2C) were co-transformed in AH109 cells with pGADT7-p53 to discreetly identify the PAH domains crucial for interaction with hp53. Yeast two hybrid results indicated that only Sin3B_168–399_ interacts with hp53 (Figure 2C). Closer examination of the three hSin3B constructs that gave positive interaction with p53 in yeast two hybrid assays, show that the overlapping amino acids between the three constructs are amino acids 193–399. This region of hSin3B contains partial PAH 2 and full PAH 3 domains of hSin3B (Figure 2C). However, β-galactosidase activity in the cell extracts reveal that while the construct containing PAH 1–3 domains of hSin3B gives a 9.9±1.813 fold increase in the relative β-galactosidase activity, the Sin3B construct containing only PAH 2–3 domains shows a mere 1.9±0.107 fold increase for hp53-Sin3B interaction (n = 3 biological replicates; Figure 2D). These observations strongly suggest that while amino acids 193–399 of hSin3B are indispensable for interaction with hp53; the hSin3B region containing the PAH 1 domain (amino acids 1–179) also contributes significantly to the overall strength of interaction with hp53 protein.

### Sin3B interaction domain (SID) of p53 lies within N-terminal 108 amino acids

In our initial yeast two hybrid assays, N-terminal deleted murine p53 (mp53 lacking the 1–72 amino acids), failed to interact with Sin3B (Supplementary Figure S5). These results and the fact that mp53 is homologous to hp53 gave us a clue that like Sin3A [20], Sin3B may also interact with the N-terminal region of p53. To confirm this hypothesis amino acids 1–108 of hp53 were cloned in pGADT7 and tested for interaction with hSin3B. As indicated in figure 2E a positive interaction was observed, suggesting that the Sin3 interaction domain (SID) of hp53 lies within N-terminal 108 amino acids.

### Increased expression of hSin3B upon treatment with Adriamycin: a DNA damaging agent

**Figure 3:**
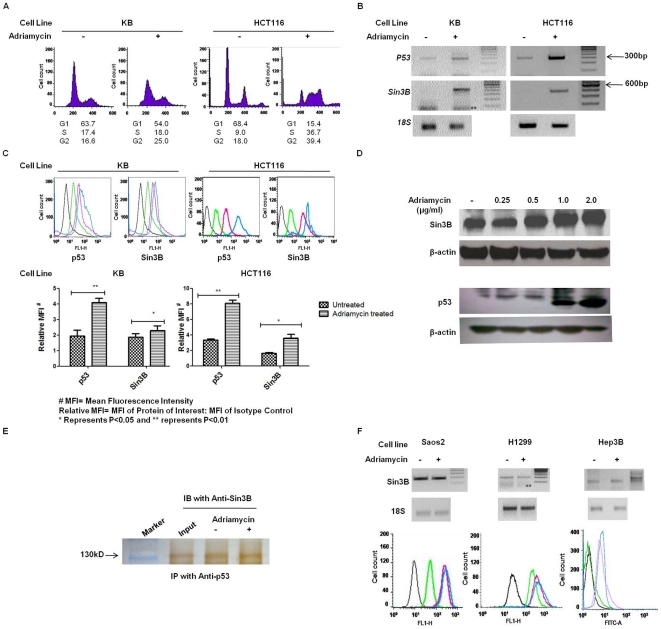
Up-regulation of hSin3B in response to Adriamycin is p53-dependent. (A) KB and HCT116 cells were treated with 1.0 µg/ml Adriamycin for 16 hours followed by propidium iodide staining and cell cycle analysis. Adriamycin treatment induced a predominant G2 cell cycle arrest in KB cells and S/G2 arrest in HCT116 cells. (B) Total RNA was isolated and cDNA was synthesized from KB and HCT116 cells with or without Adriamycin treatment. Semi-quantitative PCR results indicated increased levels of p53 and hSin3B mRNA levels in Adriamycin treated cells. (C) Upper panel shows the results of immuno-fluorescence assays using flow cytometry. Lower panel is a plot of the above results comparing the mean fluorescence intensity for p53 and hSin3B in the untreated and Adriamycin treated cells. A significant increase in p53 (P = 0.0049 in KB and P = 0.0036 in HCT116 cells) and hSin3B proteins (P = 0.0234 in KB and P = 0.0365 in HCT116 cells) was observed following Adriamycin treatment. The values have been plotted with ±SEM calculated from three (n = 3) independent experiments. (D) Western analysis of cell lysates of control and Adriamycin treated KB cells showed an increase in the hSin3B and p53 protein levels upon treatment with 1.0 and 2.0 µg/ml Adriamycin. (E) IP-Western analysis of KB cell extract after treatment with 1.0 µg/ml Adriamycin indicates the co-immunoprecipitations of hSin3B with p53 both before and after Adriamycin treatment. (F) Results of semi-quantitative PCR (upper panel) and immuno-fluorescence assays using flow cytometry (lower panel) showed no significant change in the expression levels of either hSin3B transcript or protein in p53-null cells viz. (i) Saos2 (ii) H1299 and (iii) Hep3B cells following treatment with 1.0 µg/ml Adriamycin. In all the immuno-fluorescence experiments using flow cytometer (C & F) pink histograms represent cells not treated with Adriamycin and Blue histogram represent Adriamycin treated cells. Black and green histograms represent the autofluorescence and isotype controls respectively. For all the RT-PCR experiments 18S rRNA was used as endogenous control and for western blotting, expression of β-actin was used as loading control. Representative results of three independent experimental sets are shown. In panel B and F ** indicates primer dimers.

Various cellular stresses are known to increase the levels of p53 and trigger diverse regulatory response pathways. However, there is little understanding of the regulation of levels and function of hSin3B under similar conditions of cellular stress. One such report by Grandinetti and co-workers shows an increase in the expression of Sin3B upon oncogenic stress [21]. Since we observed an interaction between p53 and hSin3B, we investigated the changes in the RNA and protein levels of hSin3B upon treatment with Adriamycin, a DNA damaging agent known to increases the levels of p53 and elicit a p53 response [22]–[24]. Similar to previous reports [25], [26], we also observed Adriamycin induced cell-cycle perturbation (one of the p53-regulated responses), wherein a predominant G2 cell cycle arrest was evident in KB cell line while a S/G2 arrest was observed in HCT116 cells (Figure 3A). Semi-quantitative RT-PCR indicated a significant increase in the RNA levels of both p53 and hSin3B post-adriamycin treatment (Figure 3B). Immuno-staining using flow cytometry and western blotting carried out under these conditions clearly show significantly higher levels of p53 as well as hSin3B proteins (Figure 3C and D). Increased expression of hSin3B, prompted us to investigate whether Adriamycin treatment results in increased interactions between p53 and hSin3B. Co-immunoprecipation assays performed in cell lysates subsequent to Adriamycin treatment show that while hSin3B and p53 interaction is maintained under conditions of stress, the increased level of hSin3B protein in cells does not lead to increased amounts of Sin3B in the p53 immune complexes (Figure 3E). To investigate whether this increase in Sin3B protein is a direct effect of DNA damage induced by Adriamycin or downstream to p53 activation, we analyzed Sin3B levels in p53 null cell lines Saos2, H1299 and Hep3B. While Adriamycin treatment induced a predominant S/G2 phase arrest in all the p53-null cell lines (Supplementary Figure S6), no increase was observed in the RNA or protein levels of Sin3B (Figure 3F).

### P53 and Sin3B/HDAC1 are recruited on the promoters of Heat shock protein 71 (*HSPA8*), Mitotic arrest deficient-like 1 protein (*MAD1*) and Zeta crystallin (*CRYZ*)

**Figure 4:**
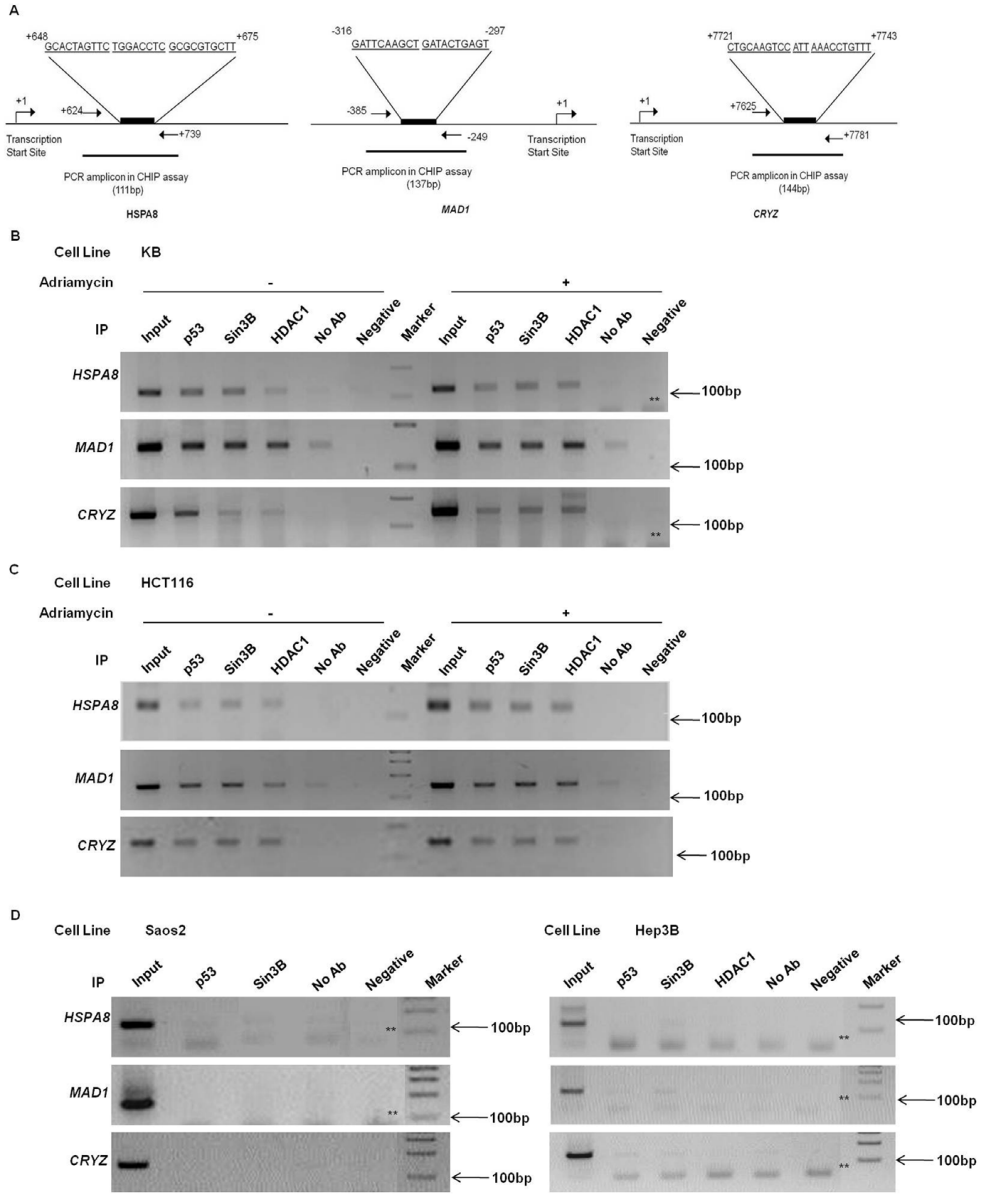
Human p53 and Sin3B/HDAC1 complex associates *in vivo* with *HSPA8*, *MAD1* and *CRYZ* promoters. (A) Schematic representation of p53 response element and the amplified promoter region of *HSPA8*, *MAD1* and *CRYZ* genes. The arrows indicate the position of the respective Forward and Reverse primers used in the ChIP Assays. (B)–(D) ChIP assays in KB (B), HCT116 (C) and p53-null cells (D). Equal amounts of cross-linked chromatin were pre-cleared and incubated with anti-p53 (sc-6243), anti-Sin3B (sc-768X) or anti-HDAC1 (sc-8410) as indicated above each lane. Following DNA precipitation samples were analyzed by PCR using primers specific for *HSPA8*, *MAD1*, *CRYZ* promoters. For negative PCR control, template was replaced with PCR-grade water. ** indicates primer dimers or non-specific amplification. Input corresponds to 10% of the total chromatin used for each immunoprecipitation. Representative figure of four independent experiments.

p53 has been found to interact with transcriptional co-repressor Sin3A in multiprotein complex which represses the transcription of many genes [5], [6]. To investigate the functional relevance of the interaction between p53 and hSin3B proteins, we explored the interactions of these two proteins with endogenous p53-responsive promoters by ChIP assays using antibodies specific for p53, Sin3B, HDAC1 in KB and HCT116 cell lines (Figure 4). A consistent recruitment of hSin3B was observed at three p53-repressed promoters and significantly high levels of endogenous *HSPA8*, *MAD1* and *CRYZ* promoters were detected in ChIP analysis in contrast to mock immunoprecipitates, both before and after Adriamycin treatment (Figure 4B and C). Sin3B-immunoprecipitates from p53-null cells (Saos2 and Hep3B) either did not contain any detectable amount of chromatin or had chromatin equivalent to mock immunoprecipitates (no antibody controls), suggesting no significant association with target promoters (Figure 4D). This suggests that recruitment of hSin3B to target promoters is p53-dependent.

### *HSPA8*, *MAD1* and *CRYZ* promoters are transcriptionally repressed upon treatment with Adriamycin

**Figure 5:**
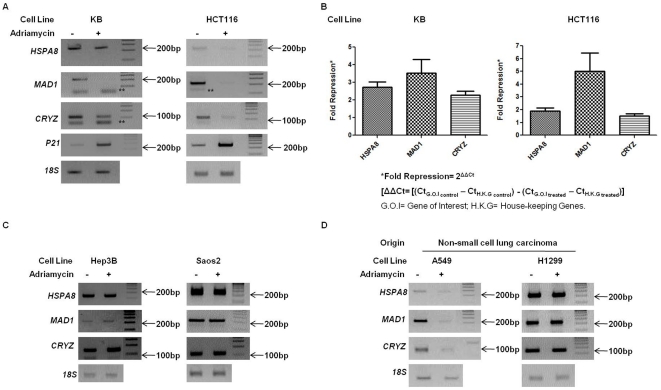
*HSPA8*, *MAD1* and *CRYZ* promoters are repressed upon treatment with Adriamycin in p53^+/+^ cells. (A & B) Total RNA was isolated and cDNA was synthesized from p53^+/+^ cell lines: KB & HCT116 with or without Adriamycin treatment. Semi-quantitative PCR results (A) indicated an Adriamycin treatment induced transcriptional repression of *HSPA8*, *MAD1* and *CRYZ* promoters and transcriptional activation of *p21*. Quantitative RT-PCR (B) re-confirmed the repression of the three genes in both KB and HCT116 cell lines. The values have been plotted with ±SEM calculated from three (n = 3) independent experiments. (C) cDNA was synthesized from total RNA isolated from p53^−/−^ cell lines: Hep3B and Saos2 cells with or without Adriamycin treatment followed by semi-quantitative PCR. No significant change in transcript levels of *HSPA8*, *MAD1* and *CRYZ* was observed in Hep3B and Saos2 cells. (D) cDNA was synthesized from total RNA isolated from two non-small cell lung carcinoma cell lines, A549 (p53^+/+^) and H1299 (p53^−/−^). No significant change in the expression was observed for the three genes in H1299 cells. In contrast a strong repression of *HSPA8*, *MAD1* and *CRYZ* was observed in A549 cells. For all expression studies 18S rRNA was used as endogenous control. Representative figures of three independent experiments are shown. ** indicates primer dimers.

We next investigated the effect of Adriamycin treatment on the mRNA levels of *HSPA8*, *MAD1* and *CRYZ* genes. Levels of *p21* transcript, a well-known p53-transactivated target, post-adriamycin treatment were also tested. The drug induced a significant reduction in the levels of *HSPA8*, *MAD1* and *CRYZ* transcripts in p53^+/+^ KB and HCT116 cells (Figure 5A). Three independent experiments (n = 3) of quantitative PCR demonstrated a 2.7±0.1696 fold repression of *HSPA8*, 3.5±0.4561 fold reduction of *MAD1* and 2.3±0.1292 fold repression of *CRYZ* transcripts, while a 29±0.6124 fold activation of *p21* transcript was evident in KB cells. Similarly in HCT116 cells, 1.9±0.2496 fold repression was observed for *HSPA8* while a 5.0±1.456 fold and 1.5±0.1670 fold repression was observed for *MAD1* and *CRYZ* genes respectively (Figure 5B and Supplementary Figure S7). Since we observed differences in Sin3B recruitment at the p53-target promoters, between p53^+/+^ and p53^−/−^ cells, we analyzed the *HSPA8*, *MAD1* and *CRYZ* transcript levels in p53^−/−^ cells as well. Although Adriamycin treatment induced predominantly S phase arrest in p53-null cells (Supplementary Figure S6), repression of these promoters was not observed (Figure 5C). Since p53 functions are known to be modulated in a cell-type specific manner (reviewed in ref. [27]), we compared the *HSPA8*, *MAD1* and *CRYZ* transcript levels, post-adriamycin treatment, in two non-small cell lung carcinoma cell lines viz. A549 (with wild-type p53 status) and H1299 (p53-null cells). Similar to our observations in KB and HCT116 cell lines, a significant repression of target genes was observed upon Adriamycin treatment in A549 cell line, while no change in the transcript levels was observed in H1299 cells (Figure 5D). Taken together, these results indicate that a ubiquitous p53-mediated recruitment of Sin3B-HDAC1 complex is indispensable for repression of *HSPA8*, *MAD1* and *CRYZ* promoters.

### H3K9 residue is hyper-methylated at *HSPA8* and *CRYZ* promoters post Adriamycin treatment

**Figure 6:**
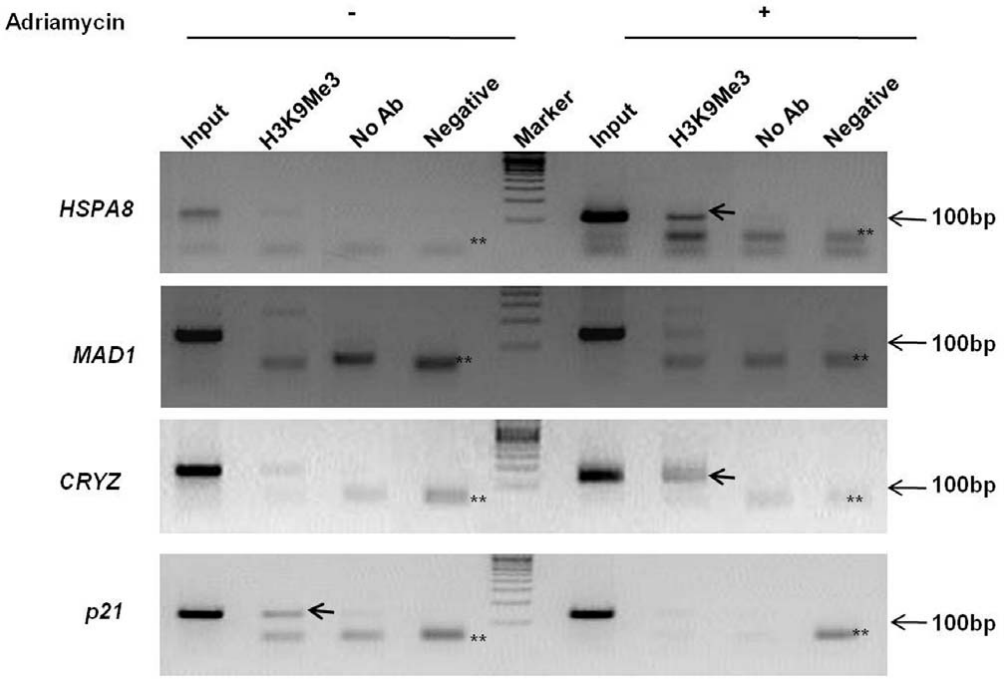
H3 Lysine 9 residue at the *HSPA8* and *CRYZ* promoters is hyper-methylated upon Adriamycin treatment. KB cells with or without Adriamycin treatment (1 µg/ml) for 16 hours were harvested. Equal amounts of cross-linked chromatin were pre-cleared and incubated with anti-H3K9Me3 antibody. Following DNA precipitation samples were analyzed by PCR using primers specific for *HSPA8*, *MAD1*, *CRYZ* promoters. For negative PCR control, template was replaced with PCR-grade water. Input corresponds to 10% of the total chromatin used for each immunoprecipitaion. ** indicates primer dimers. Arrows indicate the desired amplicon.

Since we found that p53-Sin3B are recruited at the target promoters and bring about gene repression, we next examined the epigenetic modifications at the promoter of *HSPA8*, *MAD1* and *CRYZ* before and after genotoxic insult. Methylation of H3 lysine 9 residue (H3K9) is one of the most well-characterized histone modifications and is an epigenetic marker for trans-repression [28]–[30]. We therefore analyzed the methylation at the H3K9 residue at the respective promoters in the presence and absence of Adriamycin treatment by ChIP analysis using antibodies specific for trimethylated H3K9 residue (H3K9Me3). Adriamycin clearly induced hyper-methylation of H3K9 at the promoters of p53 repressed genes, *HSPA8* and *CRYZ* while p53-activated promoter, *p21* showed hypomethylation (Figure 6). However, we did not observe any significant changes in the methylation of H3K9 residue at the *MAD1* promoter.

## Discussion

Genome wide expression analysis using micro-array has suggested that p53 can repress several cellular promoters involved in diverse pathways [3]. Some of the factors that may affect p53 transcription functions could be location of p53 binding site at the target gene and/or its proximity with the binding site of other activators or repressors, type of cellular stress and interaction with cell-type specific trans-acting factors. Diverse mechanisms have been proposed for p53 mediated repression of the target gene expression. For instance, p53 represses the alpha-fetoprotein gene expression by inhibiting the binding of hepatic nuclear factor3 on the promoter, while trans-repression by p53 on human immediate early response gene X-1 promoter is dependent on non-competitive DNA binding between p53 and Sp1 to their sites [31], [32]. An association between p53 and evolutionary conserved Sin3A co-repressor complex that lead to repression of cytoskeletal genes *Map4* and *stathmin* has also been demonstrated [6]. It is now established that p53 negatively regulates several genes like Map4, DNA topoisomerase IIα, bcl2, presenilin-1, Hsp90β, and survivin (reviewed in ref. [5]), that has put p53 in the league of bonafide transcription repressors.

In the present study we report for the first time utilization of hSin3B by p53 for its trans-repression functions. While we demonstrated a direct protein-protein interaction between p53-Sin3B *in situ* using yeast two hybrid analysis and co-immunoprecipitation, we also identified the interaction domains of the two proteins and provide evidence that the Sin3-interaction domain (SID) of p53 lies within its N-terminal 108 amino acids (Figure 2E). Interestingly, this region of p53 contains the proline rich domain, which has been associated with p53 regulatory responses like trans-repression and apoptosis [33]–[35]. Our findings also establish that amino acids 193–399 of Sin3B are essential for interaction with p53. The results of protein-protein interaction studies led us to investigate the physiological relevance of p53-Sin3B interaction. Since, p53 regulatory responses are elicited principally under conditions of stress, we investigated the status of interaction between these two proteins following exposure of cells to genotoxic stress caused by Adriamycin which functions by DNA intercalation and complex formation with topoisomerase II [22]. In previous reports an increase in the p53-Sin3A immune complex has been observed under conditions of cellular stress [6], [36]. In contrast our co-immunoprecipitation experiments did not show any significant increase in the p53-Sin3B complex. Grandinetti and co-workers have shown that levels of Sin3B are up-regulated upon oncogenic stress [21]. Our data presented here, indicate that the levels of Sin3B are also up-regulated upon genotoxic stress induced by Adriamycin, emphasizing on the potential role of Sin3B in DNA damage response pathways. Although Sin3B is expressed in p53-null cell lines, the levels of Sin3B are not modulated in these cell lines upon genotoxic stress, thus suggesting that p53 may directly or indirectly regulate Sin3B expression under conditions of stress (Figure 3F). This regulation can exist both at the transcriptional and post-transcriptional level. Since the promoter of Sin3B has not been identified, at present it cannot be ascertained whether p53 regulates Sin3B at the transcriptional level. Studying the post-transcriptional regulation of Sin3B however, requires due attention.

To analyze the recruitment of Sin3B on p53-repressed promoters, we initially screened a subset of p53-target genes that are involved in cell cycle, apoptosis and DNA repair. Our ChIP data suggested ubiquitous recruitment of Sin3B/HDAC1 on three p53-repressed promoters viz *HSPA8*, *MAD1* and *CRYZ.* Previous studies have suggested a dynamic binding of p53 to its target sites before and after stress/DNA damage [37]–[39]. Consistent with these finding, we also show recruitment of p53 and Sin3B at the p53-target sites both before and after Adriamycin treatment. ChIP and RT-PCR assays in p53^+/+^ and p53^−/−^ cell lines indicate that recruitment of hSin3B on *HSPA8*, *MAD1* and *CRYZ* is p53-dependent, and in the absence of p53 protein, no other DNA-binding transcription factors can rescue the p53-mediated repression of *HSPA8*, *MAD1* and *CRYZ* subsequent to Adriamycin treatment.

*HSPA8* has been identified as a p53-repressed target; however the mode of transcriptional repression by p53 was unclear [3]. Our results presented here clearly indicate a direct sequence-specific binding of p53 to the *HSPA8* promoter followed by p53-dependent recruitment of Sin3B/HDAC1 co-repressor complex as well as hyper-methylation of the H3K9 residue upon Adriamycin treatment. Hspa8 repression is critical for the functional activation of p53 because Hspa8 protein is known to antagonize the p53 nuclear localization by masking the NLS sequence of p53 [40].

Zeta crystallin has been shown to stabilize the mRNA of an anti-apoptotic gene, *bcl2* by binding to its unique AU rich elements (AURE) in the 3′ UTR of the mRNA [41]. In this study we observed that repression of *CRYZ* was p53-dependent and mediated through the recruitment of Sin3B/HDAC1 co-repressor complex coupled with hyper-methylation of the H3K9 residue. p53 is known to negatively regulate expression of bcl2 through a pathway independent of Sin3/HDAC1 [42]. Our results here suggest that p53 may down-regulate bcl2 expression by repression of *zeta crystallin*; latter altering the stability of *bcl2* mRNA. This hypothesis is supported by the fact that trans-repression activity of p53 is required for induction of apoptosis as mutants of p53 which are incapable of inducing apoptosis are also defective in trans-repression activity [43]. Furthermore, deletion of proline-rich domain of p53 (a region important for interaction with hSin3B as shown by us), causes loss of repression as well as induction of apoptosis without affecting its trans-activation [33]–[35]. Based on our studies we envisage that p53 may regulate apoptosis by modifying bcl-2 expression using multiple pathways.

Mitotic Arrest Deficient-like 1 Protein (MAD1/MAD1L1) is an important member of the Spindle Assembly Complex and was shown to be repressed via chromatin remodeling induced by p53-mediated recruitment of Sin3A/HDAC1 complex [7]. In the present study we observed a strong repression of *MAD1* expression upon treatment with Adriamycin that was additionally associated with the recruitment of hSin3B on p53 response element of the *MAD1* promoter. However, we did not observe H3K9 hyper-methylation on the *MAD1* promoter reflecting that the nature and combination of the histone post-translational modifications may not be same for all p53-repressed promoters. Possibly other epigenetic markers like methylation at H3K27, H4K20, ubiquitination or sumoylation may be directing the transcription at the *MAD1* promoter. Interestingly the p53 response element for *MAD1* gene is upstream of the transcription start site (TSS) while that for *HSPA8* and *CRYZ* is down-stream to the TSS (Figure 4A). The functional significance of this observation, if any, with histone methylation pattern is not clear at present.

The coordinated and dynamic regulation by specific Histone lysine methyl transferases (HKMTs) and Histone lysine demethylases (HKDMs) is an important epigenetic mechanism that plays a vital role in eukaryotic gene expression [44]. In the present study we establish an increased H3K9 trimethylation following Adriamycin treatment at the p53/Sin3B-repressed *HSPA8* and *CRYZ* promoters. H3K9 methylation is also associated with p53 mediated repression of alpha-fetoprotein via SnoN corepressor [45]. However the mechanisms dictating H3K9 methylation and the ensuing gene repression still remain unclear. H3K9 methylation provides the binding site for the HP-1 protein that participates in silencing gene expression both in the euchromatin and heterochromatin [46]. Recently it was suggested that Sin3B may co-ordinate the recruitment of HKMT and HP1protein to the E2F promoters [21]. In 2003, Yang and coworkers had established the interaction between the H3K9 methyl transferase enzyme ESET (ERG-associated protein with SET domain) and the Sin3/HDAC complex [12]. It was also recognized that histone and DNA methylation cooperate to establish long-term states of transcriptional regulation and the MeCP2 protein (methyl-CpG-binding domain protein) associates with the H3K9-specific HKMT activity [47]. The MeCP-2 protein itself is an important member of the Sin3 corepressor complex [14]. Taken together it can be speculated that ESET could be responsible for the H3K9Me3 activity observed at the *HSPA8* and *CRYZ* promoters and in concert with the MeCP2, the p53-Sin3B complex mediates the repression of the aforementioned promoters. However the role of other H3K9MTs like Suv39H1/2, G9a, GLP and Eu-HMTase cannot be ruled out. Additionally different degrees of lysine methylation (mono-, di- or trimethyl moieties) can have different functional consequences [48] and hence need to be further investigated along with an analysis of the interplay between H3K9 methylation and other repressive or activating lysine methylations (H3K27 and H3K4 respectively) in the presence and absence of genotoxic stress.

In conclusion our studies demonstrate that the transcriptional control of *HSPA8*, *MAD1* and *CRYZ* is dictated by p53 mediated recruitment of hSin3B/HDAC1 co-repressor complex and is modulated by context dependent epigenetic modifications. It is worth noting that p53 regulatory responses are dependent on type of cellular stress and can be cell-type specific. Although in our present study we have used cell lines of varied origin and demonstrated that p53-mediated repression of *HSPA8*, *MAD1* and *CRYZ* is conserved between different cellular backgrounds yet the role of cell-type specific trans-acting factors in the fine tuning of the expression of these promoters, under different cellular stresses cannot be ruled out. Our findings here propose the existence of pre-assembled p53-Sin3B co-repressor complex at the target promoters which upon cellular stress direct chromatin remodeling and downstream target gene repression. The present study highlights for the first time the essential role of Sin3B as an important associate of p53 in mediating the cellular responses to stress and in the transcriptional repression of genes encoding for heat shock proteins or proteins involved in regulation of cell cycle and apoptosis.

## Materials and Methods

### Cell culture

Head and Neck squamous carcinoma cell line KB; Human embryonic kidney cell line HEK293; Colon carcinoma cell line HCT116; non-small cell lung carcinoma cell lines, A549 and H1299; Osteosarcoma cell line, Saos2; and hepatoma cell line Hep3B; were maintained in DMEM high glucose media supplemented with 10% heat inactivated fetal bovine serum (FBS) and 60 µg/ml penicillin and 50 µg/ml streptomycin. All cells were grown at 37°C in 5% CO_2_ humidified atmosphere. All the cell lines except HCT116 and H1299 were obtained from the cell repository at NCCS, Pune. HCT116 and H1299 cells were a kind gift from the laboratory of S. Das, National Institute of Immunology, New Delhi.

### Cell treatment/p53 induction

Subconfluent (50%–60%) cultures were incubated with Adriamycin (0.25–2 µg/ml; Sigma) for 16 hr at 37°C in 5% CO_2_ humidified atmosphere. The cells were harvested for i) Western Blotting, ii) Co-immunoprecipitations, ii) Immuno-staining, iv) Chromatin Immunoprecipitation and v) RNA isolation as described below.

### Western Blotting

To assess the protein levels, cells were harvested and lysed in RIPA buffer (50 mM Tris-Cl, pH 8.0; 150 mM NaCl, 1% NP-40, 0.5% sodium deoxycholate, 2 mM EDTA, 1 mM PMSF, NaF and protease inhibitor cocktail from Sigma Aldrich) for 45 min at 4°C. The soluble protein fraction was collected by centrifugation at 12,000 rpm. Total protein was estimated using BCA protein estimation kit (Bangalore Genei; India) and equal amount of proteins (100 µg) were resolved on 8% SDS–polyacrylamide gels and transferred onto immunoblot-PVDF membranes (Santacruz Biotechnology, USA). Western blots were blocked in 3% Bovine Serum Albumin (BSA). Blots were incubated with 1 µg/ml of 1: 1000 diluted antibody (anti-p53, sc-98; anti-Sin3B, sc-13145/sc-768/sc-55516; anti-βactin, sc-47773) for 1.5 hr at room temperature, followed by washing in PBS containing 0.5% Tween-20. Thereafter the blot was incubated in peroxidase-conjugated secondary antibody of 1∶5000 dilution (Santacruz Biotechnology, USA) and detected by using DAB (3,3′-diaminobenzidine tetrahydrochloride, Bangalore Genei; India) substrate or by chemiluminescence detection (Santacruz Biotechnology, USA).

### Immunoprecipitation

Subconfluent cultures of cells were harvested and lysed in NP-40 buffer (20 mM Tris-HCl buffer pH-7.4, 100 mM NaCl, 0.5 mM EDTA, 0.5% NP-40) supplemented with protease inhibitor cocktail (Sigma Aldrich). Total protein (2 mg) from each sample was immunoprecipitated with 1 µg of desired antibody. Each immunoprecipitate was washed thrice in NP-40 buffer, fractionated on 8% SDS-PAGE and transferred overnight onto Immuno-Blot PVDF membrane (Santacruz Biotechnology, USA) followed by western blotting as described above. ImmunoCruz™ IP/WB Optima E System (sc-45042) from Santa Cruz was used for performing the reciprocal CoIP experiments that involved IP with mouse anti-Sin3B (sc-13145) and detection by anti-p53 (sc-98). IP/WB Optima E System is intended for use in such homologous IP/WB applications and is optimized to detect the desired Western blot probe antibody without detection of heavy and light chains of the IP antibody.

### Yeast two Hybrid assays

Yeast two hybrid tests were performed using the Matchmaker Two Hybrid System 3 (Clontech, USA) according to the manufacturer's protocols. Yeast (*Saccharomyces cerevisiae*) strain AH109 was used to determine protein-protein interactions. The GAL4 DNA binding domain (BD) vector pGBKT7 and the activation domain (AD) vector pGADT7 were used throughout. Desired DNA fragments containing coding sequence of human p53 (Gene ID: 7157) and human Sin3B (Gene ID: 23309) were PCR amplified from human brain cDNA library (Clontech) using gene-specific primers (Supplementary Table S1). Full length human p53 was cloned in the pGADT7 vector (pGADT7-hp53). Three overlapping fragments of human Sin3B (spanning the full length coding region; Supplementary Figure S3) were cloned in pGBKT7 vector: pGBKT7-Sin3B_1–399_ (N-terminal Sin3B amino acids 1–399), pGBKT7-Sin3B_193–468_ (amino acids 193–468) and pGBKT7-Sin3B_442–1162_ (C-terminal amino acids 442–1162). All other truncated p53 and Sin3B constructs were derived from pGADT7-hp53 and pGBKT7-Sin3B_1–399_ respectively.

pGBKT7-Sin3B and pGADT7-hp53 clones were co-transformed in yeast strain AH109 and co-transformants were selected using drop-out (DO) medium, lacking tryptophan and leucine (SD LT). The selected co-transformants were replica plated on to dropout medium lacking leucine, tryptophan, adenine, histidine and containing Xgal (SD QDO-Xgal) and allowed to grow until the colonies appeared. AH109 cotransformed with murine pGBKT7-p53/pGADT7-T (supplied by Clontech) was used as positive control, whereas yeast cotransformed with pGADT7/pGBKT7 were used as negative control. β-galactosidase assay was performed as per the manufacturer's protocol.

### Expression Analysis of Human p53 and Human Sin3B

Monolayer cells were washed twice with ice cold PBS and trypsinized. The trypsinized cells were washed with ice cold wash buffer (PBS +0.1% BSA). The cells were then fixed with 2% Paraformaldehyde (PFA) at a density of 10^6^ cells/100 µl and stored overnight at 4°C. The cells were washed twice with ice cold wash buffer to completely remove PFA. The cells were incubated in permeabilization buffer (0.5% saponin +0.05% Triton X 100 in PBS) for 10 min in ice and then washed twice with ice cold wash buffer. The cells were then incubated with 1–2 µg of monoclonal antibody (anti p53 or anti Sin3B) diluted in dilution buffer (PBS+0.01% saponin +1%BSA +1% sodium azide) and incubated at 4°C for one and half hours. The cells were washed twice with ice cold wash buffer and then incubated with FITC conjugated secondary antibody for 45 minutes in dark at 4°C. The cells were washed twice with ice cold wash buffer and analyzed for expression of hp53 or Sin3B on FACSCalibur and LSR II using CellQuestPro and FlowJo Softwares (Becton Dickinson).

### Chromatin Immunoprecipitation

Cultured cells were crosslinked using 1% formaldehyde, lysed, sonicated and samples were immunoprecipitated, washed and reverse crosslinked as described by Soutoglou and Talianidis in 2002 with several modifications [49]. Briefly, the crosslinked cells were suspended in 0.1% SDS lysis buffer (50 mM Tris pH 8.0, 140 mM NaCl, 1 mM EDTA, 1% TritonX100, 0.1% Sodium deoxycholate, 0.1% SDS, Protease inhibitors cocktail) and then sonicated for 30 cycles of 30s pulse at maximum power using a Bioruptor (Diagenode) to an average length of 200–500 bp of DNA. After centrifugation, the samples were precleared with Protein A-Agarose beads (preblocked with 1 mg/ml salmon sperm DNA and 1 mg/ml of BSA). The precleared chromatin was immunoprecipitated with 1–2 µg of antibodies, and the immune complexes were collected by adsorption to Protein A-Agarose. The beads were washed thrice with 0.1% SDS lysis buffer, twice each with wash buffer A (50 mM TrisCl pH 8.0, 500 mM NaCl, 1 mM EDTA, 1% TritonX 100, 0.1% Sodium deoxycholate, 0.1% SDS and protease inhibitor cocktail), wash buffer B (20 mM TrisCl pH 8.0, 250 mM LiCl, 1 mM EDTA, 0.5% NP-40, 0.5% Sodium deoxycholate and protease inhibitor cocktail) and 1× TE. The immunocomplexes were eluted with 1%SDS, 0.1 M NaHCO_3_ at 37°C for 30 min; decrosslinked by adding 200 mM NaCl and incubated at 65°C for 6 hr. After successive treatments with 10 µg of Rnase A and Proteinase K (20 µg/ml), the samples were extracted with phenol-chloroform and precipitated with ethanol. PCR of the target promoter were performed on immunoprecipitated chromatin using promoter specific primers (Supplementary Table S2). ChIP DNA was detected by ethidium bromide staining of PCR products after gel electrophoresis.

### Total RNA isolation and semi-quantitative and quantitative RT-PCR

Total RNA was extracted with RNeasy plus Mini kit (Qiagen, USA). RNA (1 µg) was reverse transcribed with first strand cDNA synthesis kit (Fermentas). Gene-specific primers designed across the exon-exon boundary were used for RT-PCR (Supplementary Table S3). Quantitative RT-PCR (qRT-PCR) was performed in triplicates using SYBER green quantitative PCR kit (Eurogenetec, Germany) and a 7300 detector (Applied Biosystems) under conditions standardized for each primer set. PCR quantification was done using the comparative Ct method (delta delta C_t_ method). 18S ribosomal RNA gene was used as endogenous control.

### Statistical Analysis

All statistical analysis was performed using GraphPad Prism version 5 (GraphPad Software Inc., San Diego, CA, USA). The results of flow cytometry and qRT-PCR are presented as mean ± SEM. Paired t-test was used for comparisons and calculating the level of significance.

## Supporting Information

Figure S1 **IP-Western analysis of KB cell extract with different antibodies specific for hSin3B.** KB cell lysates were immunoprecipitated with anti-p53 antibody (sc-98, Santa Cruz Biotechnology, USA) followed by immunoblotting (IB) with different antibodies specific for Sin3B (sc-13145, sc-55516, sc-768, Santa Cruz Biotechnology) as indicated. Western analysis indicates the consistent presence of Human Sin3B in p53 immune complexes in KB cell extract.
(DOC)

Figure S2 **Association of HDAC1 with Sin3B immune complexes.** (**A**) KB (p53^+/+^) cell lysates were immunoprecipitated with anti-HDAC1 antibody (sc-8410, Santa Cruz Biotechnology) followed by immunoblotting (IB) with antibodies specific for Sin3B as indicated. Western analysis indicates the consistent presence of Human Sin3B in HDAC1 immune complexes. (**B**) H1299 (p53^−/−^) cell lysates were immunoprecipitated with anti-HDAC1 antibody or anti-Sin3B antibody as indicated followed by immunoblotting (IB) with appropriate antibodies (Anti-Sin3B in left panel and Anti-HDAC1 in the right panel). IP-Western analysis indicates the presence of Human Sin3B-HDAC1 immune complexes in a p53-independent manner.
(DOC)

Figure S3 **Three overlapping fragments of human Sin3B.** Sin3B_1–399_ (N-terminal Sin3B amino acids 1–399); Sin3B_193–468_ (amino acids 193–468) and Sin3B_442–1162_ (amino acids 442–1162); spanning the full length coding region of human Sin3B were cloned in yeast shuttle vector pGBKT7 vector.
(DOC)

Figure S4 **Western blot analysis for p53 and Sin3B expression in AH109 co-transformants.** (**A**) Immunoblotting to confirm the expression of hp53 in cotransformants in yeast cells. Western analysis of hp53 cotransformed with different overlapping fragments of Sin3B in AH109 cell lysates as indicated above each lane i.e. pGBKT7-Sin3B_1–399_ X pGADT7-hp53, pGBKT7-Sin3B_193–468_ X pGADT7-hp53, pGBKT7-Sin3B_442–1162_ X pGADT7-hp53 and pGBKT7 X pGADT7-hp53. (**B**) Western blot to check the expression of the three Sin3B-pGBKT7 clones expressed in AH109 cells. Western analysis of Sin3B_1–399_ (Panel i), Sin3B_193–468_ (Panel ii), and Sin3B_442–1162_ (Panel iii) in different cotransformants as indicated i.e. pGBKT7-Sin3B X pGADT7-hp53 or pGBKT7-Sin3B X pGADT7.
(DOC)

Figure S5 **Yeast two Hybrid analysis for the interaction of hSin3B with mouse p53 (mp53 lacking the N-terminal 72 amino acids).** Yeast AH109 cells were co-transformed with (i) pGBK T7-mp53 (GBK-p53) and pGAD T7-Sin3B_1–399_ (NTS) (ii) GBK vector (GBK) and NTS (iii) GBK-p53 and T antigen (GADT) as indicated on the plates. The protein-protein interactions were checked by growing the co-transformants on selective SD QDO medium (Quadruple drop-out medium lacking leucine, tryptophan, adenine and histidine). Positive interaction was observed only between pGBK-p53 and GADT antigen as indicated by the black arrow but no interaction was observed between Sin3B and N-terminal deleted mouse p53 as no growth was observed on the SD QDO medium (patches 1–16).
(DOC)

Figure S6 **Adriamycin induces a predominant S/G2 cell cycle arrest in p53 null cell lines.** Saos2, H1299 and Hep3B cells were treated with 1.0 µg/ml Adriamycin for 16 hours followed by propidium iodide staining and cell cycle analysis. Adriamycin treatment induced a predominant S/G2 cell cycle arrest in the p53^−/−^ cells.
(DOC)

Figure S7 **Levels of** ***p21*** **are up-regulated in KB and HCT116 cells in response to Adriamycin treatment.** qRT-PCR was performed to calculate fold activation of *p21* transcript. A 29±0.6124 fold transactivation in KB cells and 15.46±0.5357 fold activation in HCT116 cells were observed for *p21*.
(DOC)

Table S1 **Primer Sequences for Cloning Sin3B and p53 in yeast shuttle vectors pGBKT7 and pGADT7.**
(DOC)

Table S2 **Primers for Chromatin immunoprecipitation.**
(DOC)

Table S3 **Primers for semi-quantitative and Real time PCR.**
(DOC)
