# Supplementary material for: Tumor Suppressor Protein p53 Recruits Human Sin3B/HDAC1 Complex for Down-Regulation of Its Target Promoters in Response to Genotoxic Stress
Source: PLoS One. 2011 Oct 20;6(10):e26156. doi: 10.1371/journal.pone.0026156 (PMC3197607; doi:10.1371/journal.pone.0026156)
Supplement: Figure S1 — IP-Western analysis of KB cell extract with different antibodies specific for hSin3B. KB cell lysates were immunoprecipitated with anti-p53 antibody (sc-98, Santa Cruz Biotechnology, USA) followed by immunoblotting (IB) with different antibodies specific for Sin3B (sc-13145, sc-55516, sc-768, Santa Cruz Biotechnology) as indicated. Western analysis indicates the consistent presence of Human Sin3B in p53 immune complexes in KB cell extract. (DOC) [file pone.0026156.s001.doc]

**
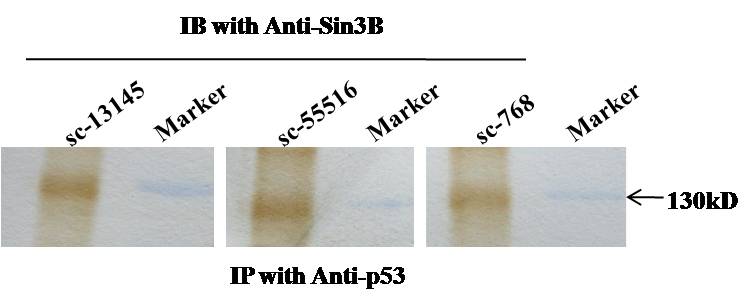
**

**Figure S1. IP-Western analysis of KB cell extract with different antibodies specific for hSin3B.** KB cell lysates were immunoprecipitated with anti-p53 antibody (sc-98, Santa Cruz Biotechnology, USA) followed by immunoblotting (IB) with different antibodies specific for Sin3B (sc-13145, sc-55516, sc-768, Santa Cruz Biotechnology) as indicated. Western analysis indicates the consistent presence of Human Sin3B in p53 immune complexes in KB cell extract.
