# Supplementary material for: Tumor Suppressor Protein p53 Recruits Human Sin3B/HDAC1 Complex for Down-Regulation of Its Target Promoters in Response to Genotoxic Stress
Source: PLoS One. 2011 Oct 20;6(10):e26156. doi: 10.1371/journal.pone.0026156 (PMC3197607; doi:10.1371/journal.pone.0026156)
Supplement: Figure S2 — Association of HDAC1 with Sin3B immune complexes. (A) KB (p53+/+) cell lysates were immunoprecipitated with anti-HDAC1 antibody (sc-8410, Santa Cruz Biotechnology) followed by immunoblotting (IB) with antibodies specific for Sin3B as indicated. Western analysis indicates the consistent presence of Human Sin3B in HDAC1 immune complexes. (B) H1299 (p53−/−) cell lysates were immunoprecipitated with anti-HDAC1 antibody or anti-Sin3B antibody as indicated followed by immunoblotting (IB) with appropriate antibodies (Anti-Sin3B in left panel and Anti-HDAC1 in the right panel). IP-Western analysis indicates the presence of Human Sin3B-HDAC1 immune complexes in a p53-independent manner. (DOC) [file pone.0026156.s002.doc]

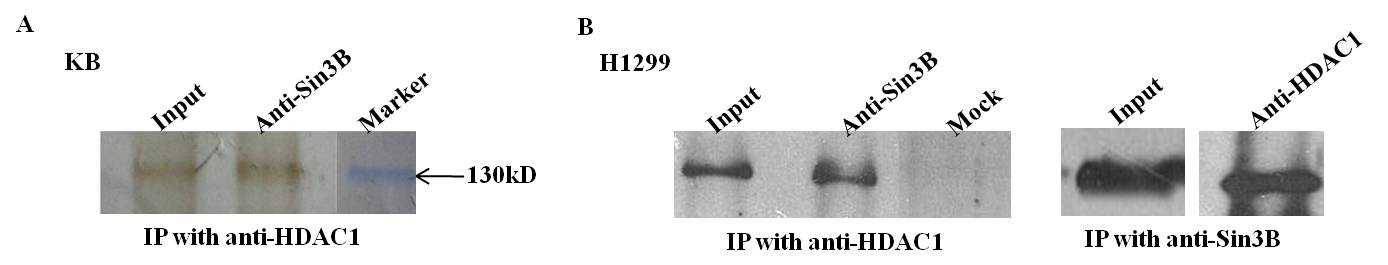


**Figure S2. Association of HDAC1 with Sin3B immune complexes. (A)** KB (p53^+/+^) cell lysates were immunoprecipitated with anti-HDAC1 antibody (sc-8410, Santa Cruz Biotechnology) followed by immunoblotting (IB) with antibodies specific for Sin3B as indicated. Western analysis indicates the consistent presence of Human Sin3B in HDAC1 immune complexes. **(B)** H1299 (p53^-/-^) cell lysates were immunoprecipitated with anti-HDAC1 antibody or anti-Sin3B antibody as indicated followed by immunoblotting (IB) with appropriate antibodies (Anti-Sin3B in left panel and Anti-HDAC1 in the right panel). IP-Western analysis indicates the presence of Human Sin3B-HDAC1 immune complexes in a p53-independent manner.
