# Supplementary material for: Tumor Suppressor Protein p53 Recruits Human Sin3B/HDAC1 Complex for Down-Regulation of Its Target Promoters in Response to Genotoxic Stress
Source: PLoS One. 2011 Oct 20;6(10):e26156. doi: 10.1371/journal.pone.0026156 (PMC3197607; doi:10.1371/journal.pone.0026156)
Supplement: Figure S3 — Three overlapping fragments of human Sin3B. Sin3B1–399 (N-terminal Sin3B amino acids 1–399); Sin3B193–468 (amino acids 193–468) and Sin3B442–1162 (amino acids 442–1162); spanning the full length coding region of human Sin3B were cloned in yeast shuttle vector pGBKT7 vector. (DOC) [file pone.0026156.s003.doc]

**
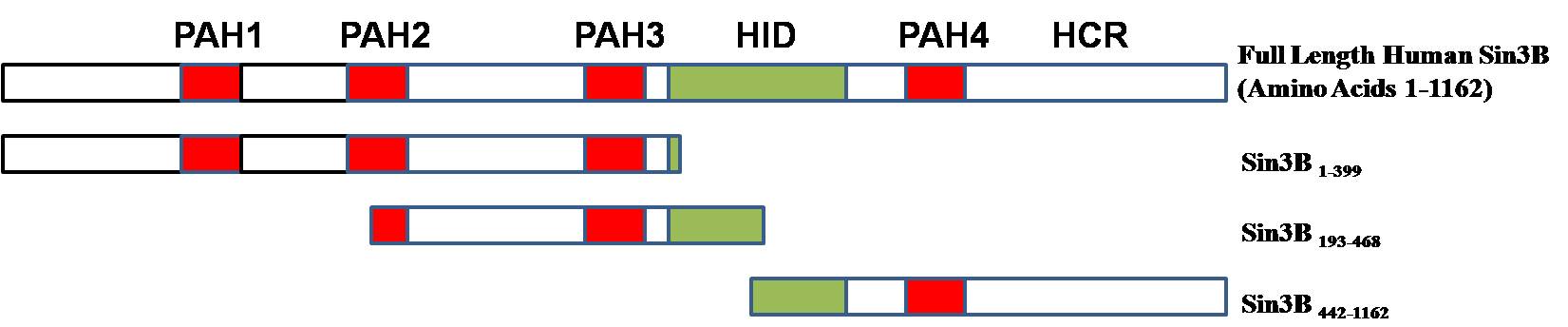
**

**Figure S3. Three overlapping fragments of human Sin3B.** Sin3B_1-399_ (N-terminal Sin3B amino acids 1-399); Sin3B_193-468_ (amino acids 193-468) and Sin3B_442-1162_ (amino acids 442-1162); spanning the full length coding region of human Sin3B were cloned in yeast shuttle vector pGBKT7 vector.
