# Supplementary material for: Tumor Suppressor Protein p53 Recruits Human Sin3B/HDAC1 Complex for Down-Regulation of Its Target Promoters in Response to Genotoxic Stress
Source: PLoS One. 2011 Oct 20;6(10):e26156. doi: 10.1371/journal.pone.0026156 (PMC3197607; doi:10.1371/journal.pone.0026156)
Supplement: Figure S4 — Western blot analysis for p53 and Sin3B expression in AH109 co-transformants. (A) Immunoblotting to confirm the expression of hp53 in cotransformants in yeast cells. Western analysis of hp53 cotransformed with different overlapping fragments of Sin3B in AH109 cell lysates as indicated above each lane i.e. pGBKT7-Sin3B1–399 X pGADT7-hp53, pGBKT7-Sin3B193–468 X pGADT7-hp53, pGBKT7-Sin3B442–1162 X pGADT7-hp53 and pGBKT7 X pGADT7-hp53. (B) Western blot to check the expression of the three Sin3B-pGBKT7 clones expressed in AH109 cells. Western analysis of Sin3B1–399 (Panel i), Sin3B193–468 (Panel ii), and Sin3B442–1162 (Panel iii) in different cotransformants as indicated i.e. pGBKT7-Sin3B X pGADT7-hp53 or pGBKT7-Sin3B X pGADT7. (DOC) [file pone.0026156.s004.doc]

**
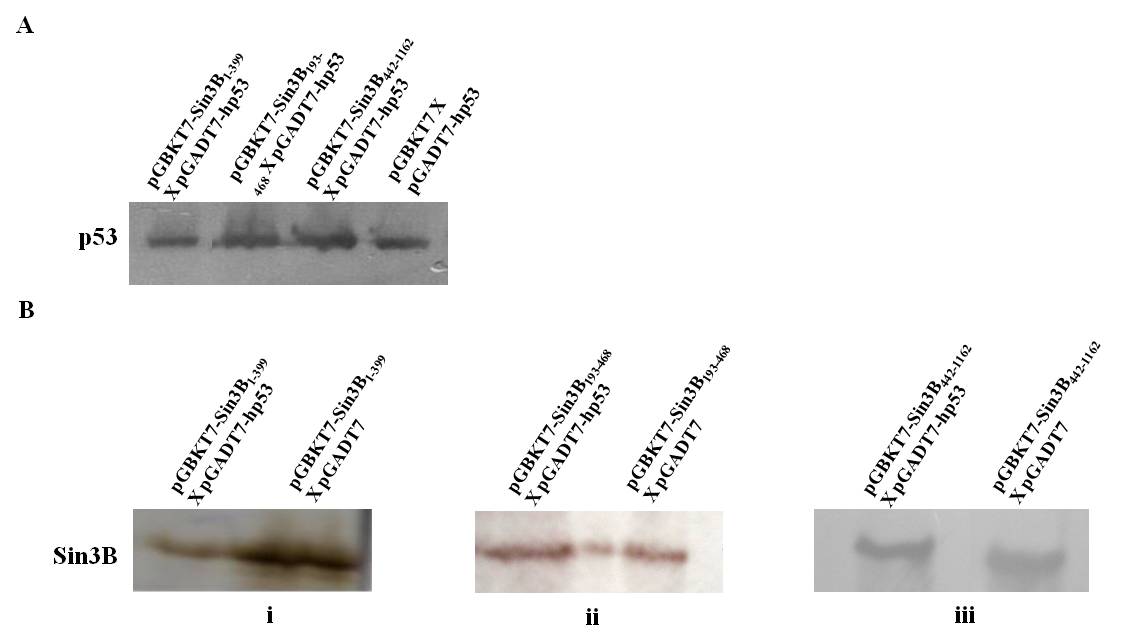
**

**Figure S4. Western blot analysis for p53 and Sin3B expression in AH109 co-transformants. (A)** Immunoblotting to confirm the expression of hp53 in cotransformants in yeast cells. Western analysis of hp53 cotransformed with different overlapping fragments of Sin3B in AH109 cell lysates as indicated above each lane i.e. pGBKT7-Sin3B_1-399_ X pGADT7-hp53, pGBKT7-Sin3B_193-468_ X pGADT7-hp53, pGBKT7-Sin3B_442-1162_ X pGADT7-hp53 and pGBKT7 X pGADT7-hp53. **(B)** Western blot to check the expression of the three Sin3B-pGBKT7 clones expressed in AH109 cells. Western analysis of Sin3B_1-399_ (Panel i), Sin3B_193-468_ (Panel ii), and Sin3B_442-1162_ (Panel iii) in different cotransformants as indicated i.e. pGBKT7-Sin3B X pGADT7-hp53 or pGBKT7-Sin3B X pGADT7.
