# Supplementary material for: Tumor Suppressor Protein p53 Recruits Human Sin3B/HDAC1 Complex for Down-Regulation of Its Target Promoters in Response to Genotoxic Stress
Source: PLoS One. 2011 Oct 20;6(10):e26156. doi: 10.1371/journal.pone.0026156 (PMC3197607; doi:10.1371/journal.pone.0026156)
Supplement: Figure S5 — Yeast two Hybrid analysis for the interaction of hSin3B with mouse p53 (mp53 lacking the N-terminal 72 amino acids). Yeast AH109 cells were co-transformed with (i) pGBK T7-mp53 (GBK-p53) and pGAD T7-Sin3B1–399 (NTS) (ii) GBK vector (GBK) and NTS (iii) GBK-p53 and T antigen (GADT) as indicated on the plates. The protein-protein interactions were checked by growing the co-transformants on selective SD QDO medium (Quadruple drop-out medium lacking leucine, tryptophan, adenine and histidine). Positive interaction was observed only between pGBK-p53 and GADT antigen as indicated by the black arrow but no interaction was observed between Sin3B and N-terminal deleted mouse p53 as no growth was observed on the SD QDO medium (patches 1–16). (DOC) [file pone.0026156.s005.doc]

**
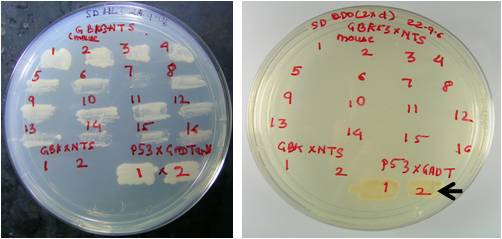
**

**Figure S5. Yeast two Hybrid analysis for the interaction of hSin3B with mouse p53 (mp53 lacking the N-terminal 72 amino acids).** Yeast AH109 cells were co-transformed with (i) pGBK T7-mp53 (GBK-p53) and pGAD T7-Sin3B_1-399_ (NTS) (ii) GBK vector (GBK) and NTS (iii) GBK-p53 and T antigen (GADT) as indicated on the plates. The protein-protein interactions were checked by growing the co-transformants on selective SD QDO medium (Quadruple drop-out medium lacking leucine, tryptophan, adenine and histidine). Positive interaction was observed only between pGBK-p53 and GADT antigen as indicated by the black arrow but no interaction was observed between Sin3B and N-terminal deleted mouse p53 as no growth was observed on the SD QDO medium (patches 1-16).
