# Supplementary material for: Tumor Suppressor Protein p53 Recruits Human Sin3B/HDAC1 Complex for Down-Regulation of Its Target Promoters in Response to Genotoxic Stress
Source: PLoS One. 2011 Oct 20;6(10):e26156. doi: 10.1371/journal.pone.0026156 (PMC3197607; doi:10.1371/journal.pone.0026156)
Supplement: Figure S6 — Adriamycin induces a predominant S/G2 cell cycle arrest in p53 null cell lines. Saos2, H1299 and Hep3B cells were treated with 1.0 µg/ml Adriamycin for 16 hours followed by propidium iodide staining and cell cycle analysis. Adriamycin treatment induced a predominant S/G2 cell cycle arrest in the p53−/− cells. (DOC) [file pone.0026156.s006.doc]

**
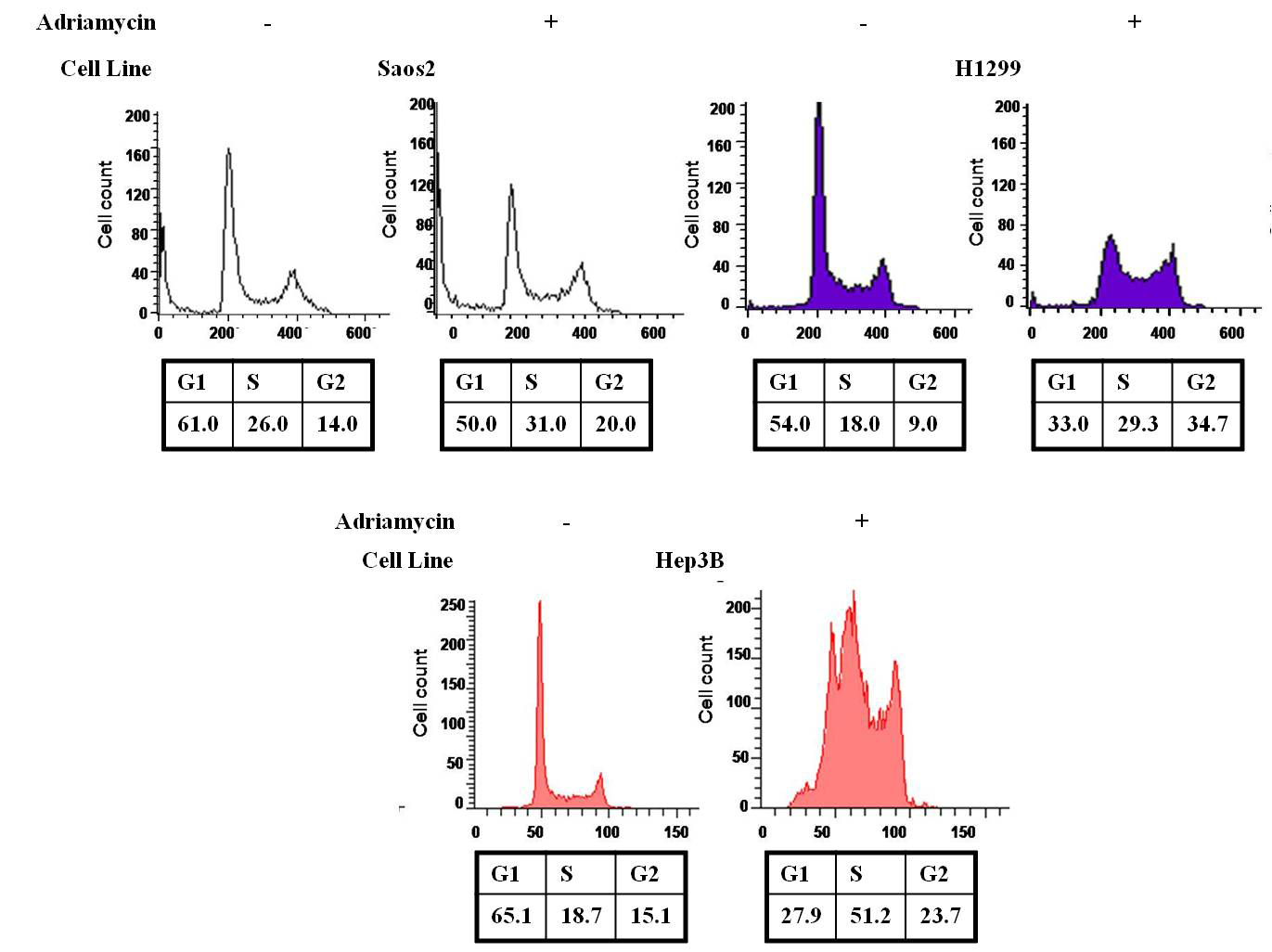
**

**Figure S6. Adriamycin induces a predominant S/G2 cell cycle arrest in p53 null cell lines.** Saos2, H1299 and Hep3B cells were treated with 1.0µg/ml Adriamycin for 16 hours followed by propidium iodide staining and cell cycle analysis. Adriamycin treatment induced a predominant S/G2 cell cycle arrest in the p53^-/-^ cells.
