# Supplementary material for: Tumor Suppressor Protein p53 Recruits Human Sin3B/HDAC1 Complex for Down-Regulation of Its Target Promoters in Response to Genotoxic Stress
Source: PLoS One. 2011 Oct 20;6(10):e26156. doi: 10.1371/journal.pone.0026156 (PMC3197607; doi:10.1371/journal.pone.0026156)
Supplement: Figure S7 — Levels of p21 are up-regulated in KB and HCT116 cells in response to Adriamycin treatment. qRT-PCR was performed to calculate fold activation of p21 transcript. A 29±0.6124 fold transactivation in KB cells and 15.46±0.5357 fold activation in HCT116 cells were observed for p21. (DOC) [file pone.0026156.s007.doc]

**
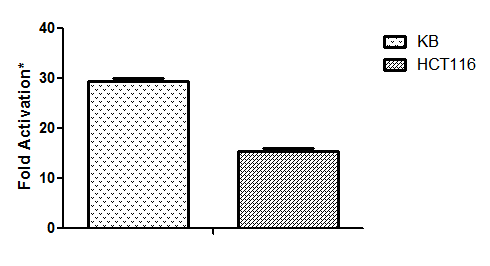
**

**Figure S7. Levels of *p21* are up-regulated in KB and HCT116 cells in response to Adriamycin treatment.** qRT-PCR was performed to calculate fold activation of *p21* transcript. A 29±0.6124 fold transactivation in KB cells and 15.46±0.5357 fold activation in HCT116 cells were observed for *p21*.
