# Supplementary material for: Tumor Suppressor Protein p53 Recruits Human Sin3B/HDAC1 Complex for Down-Regulation of Its Target Promoters in Response to Genotoxic Stress
Source: PLoS One. 2011 Oct 20;6(10):e26156. doi: 10.1371/journal.pone.0026156 (PMC3197607; doi:10.1371/journal.pone.0026156)
Supplement: Table S1 — Primer Sequences for Cloning Sin3B and p53 in yeast shuttle vectors pGBKT7 and pGADT7. (DOC) [file pone.0026156.s008.doc]

| Clone | Forward Primer (5’-3’) | Reverse Primer (5’-3’) |
| --- | --- | --- |
| pGBKT7-Sin3B_1-399_ | TGATTCTCGCATATGGCGCACGCTGGC* | CCGGTAGCTGGATCCTATGCGCTTGCA^#^ |
| pGBKT7-Sin3B_193-468_ | GCCGCGGCATATGGATTCGTACCAGAAG* | CATATGGTCGACCTGCAGGCGGCC^ |
| pGBKT7-Sin3B_442-1162_ | CCAAGCTTCATAT GTACGAGGAGCAGC | GGCGGGTCGACTGCACGCGG |
| pGBKT7-Sin3B_1-247_ | TGATTCTCGCATATGGCGCACGCTGGC* | GCCCGGATCCTGTGAACAGAGACC^#^ |
| pGBKT7-Sin3B_1-179_ | TGATTCTCGCATATGGCGCACGCTGGC* | GTGGTCTAGGATCCGGGTTTTAATCTTAT^#^ |
| pGBKT7-Sin3B_168-399_ | CTATGTGCATATGATTAAAACCCGCTTCC* | CCGGTAGCTGGATCCTATGCGCTTGCA^#^ |
| pGADT7-hp53 | CTATCGATTCACTGCCATGGA@ | GAACTCGAGAATGTCAGTCTGAG^$^ |
| pGADT7-hp53_1-108_ | CTATCGATTCACTGCCATGGA@ | CTCGAGATGACAGGGGCCAG^$^ |

All enzyme sites used for cloning are highlighted in yellow.

* Enzyme Site: NdeI

@ Enzyme Site: Cla1

# Enzyme Site: BamHI

$ Enzyme Site: XhoI

^ The insert was cloned in pGEM Teasy vector ; EcoRI site in the pGEM Teasy vector was used

for further cloning in pGBKT7.

**Table S1. Primer Sequences for Cloning Sin3B and p53 in yeast shuttle vectors pGBKT7 and pGADT7**
