# Supplementary material for: Tumor Suppressor Protein p53 Recruits Human Sin3B/HDAC1 Complex for Down-Regulation of Its Target Promoters in Response to Genotoxic Stress
Source: PLoS One. 2011 Oct 20;6(10):e26156. doi: 10.1371/journal.pone.0026156 (PMC3197607; doi:10.1371/journal.pone.0026156)
Supplement: Table S2 — Primers for Chromatin immunoprecipitation. (DOC) [file pone.0026156.s009.doc]

| Promoter | Forward Primer (5’-3’) | Reverse Primer (5’-3’) |
| --- | --- | --- |
| *HspA8* | TGGGTAGATGGGTCCTTCAT | AATAGTGCCCATCACCTCCT |
| *Mad1* | ACTGGGAAGGTAGCCTAGTAGCATA | AGCCTCCTCGGACAAACTTGC |
| *Cryz* | TCCACCATGATTGTGAGACC | CAAACATTTACCTGACACCCA |
| *P21* | GCTGTGGCTCTGATTGGCTTTC | CTGTCTCCTACCATCCCCTTCCT |

**Table S2. Primers for Chromatin immunoprecipitation**
