# Supplementary material for: Tumor Suppressor Protein p53 Recruits Human Sin3B/HDAC1 Complex for Down-Regulation of Its Target Promoters in Response to Genotoxic Stress
Source: PLoS One. 2011 Oct 20;6(10):e26156. doi: 10.1371/journal.pone.0026156 (PMC3197607; doi:10.1371/journal.pone.0026156)
Supplement: Table S3 — Primers for semi-quantitative and Real time PCR. (DOC) [file pone.0026156.s010.doc]

| Gene | Forward Primer (5’-3’)* | Reverse Primer (5’-3’)* |
| --- | --- | --- |
| *HspA8* | GCCGTTTGAGCAAGGAAGACA | CAGCAGTCTGATTCTTATCAAGCC |
| *Mad1* | CAGGGTGACTATGACCAGAGCAG | TCAGCTCTGCCACCTCCTTG |
| *Cryz* | GAGTGATAGTTGTTGGCAGCAGAG | TGCTGAAATTCCTCCTTGGTTG |
| *p21* | GACCAGCATGACAGATTTCTACCACTC | AACCTCTCATTCAACCGCCTAG |
| *p53* | AGTGTGGTGGTGCCCTATGAGC | TGGTGTTGTTGGGCAGTGCT |
| *Sin3Ba.*  *Sin3Bb.* | AGAGCATCGATACTCCTGGAG  AGATCTGGCGGGAGCAGTATGAGAA | TTGAAGTCCTCTGCACCGTC  TTCTTCCGCTCACTGGGGTCTGT |

*Primers were designed at the exon-exon junctions except primer pair Sin3B.

**Table S3. Primers for semi-quantitative and Real time PCR**
